# Normal limits of home measured spatial gait parameters of the elderly population and their association with health variables

**DOI:** 10.1038/s41598-018-31507-1

**Published:** 2018-09-04

**Authors:** Alexandra Herrero-Larrea, Antonio Miñarro, Leire Narvaiza, César Gálvez-Barrón, Natalia Gonzalo León, Esther Valldosera, Elisabet Felipe, Rosa Ana Valverde, Liane Kruse, Joan Bosch Sabater, Alejandro Rodríguez-Molinero

**Affiliations:** 1 Consorci Sanitari del Garraf, Vilanova i la Geltrú, Barcelona, Spain; 2 Department of Genetics, Microbiology and Statistics, Faculty of Biology, University of Barcelona, Barcelona, Spain; 3 Parc Sanitari Sant Joan de Déu, Centre de Salut Mental del Garraf. Vilanova i la Geltrú, Barcelona, Spain; 4 Fundació Privada Sant Antoni Abat. Vilanova i la Geltrú, Barcelona, Spain; 5 Universitat Internacional de Catalunya, Sant Cugat del Vallès, Barcelona, Spain

## Abstract

Gait studies in the elderly population have been always conducted in gait labs or spacious clinical facilities, which influence gait parameters, and also implies that the participants have to be able to move to these facilities. Indoors gait characteristics of the elderly population have been very little studied. In this study, we aim to define the normal limits of the spatial gait parameters of the elderly, when walking at home, and to analyze relationship existing between the spatial gait parameters to other health variables. For such purpose, we conducted a transversal study on a probabilistic sample of 431 Spanish community-dwelling older, in which the spatial gait parameters were recorded by using an ink footprints method. We found that the mean stride length indoors was 88.47 cm (SD:26.05 cm; mean CI95%:85.52–91.41 cm), and the mean step width was 10.34 cm (SD:4.37 cm; mean CI95%:9.84–10.83 cm). Stride length was shorter in women and the oldest group, and was significantly influenced by the strength, balance, and physical activity. Stride width was larger in the oldest group and mainly affected by balance. A composite parameter including width and normalized stride length was independent from sex, and strongly differentiated between age groups. This parameter was affected by strength.

## Introduction

Gait disorders are very prevalent in the elderly population and are important indicators of the health status of the elderly. In clinical practice, the gait has been classically evaluated by direct observation, or by quantification with simple methods such as in the case of the Up and Go test^1^ or the Tinetti scale^2^. However, this type of evaluation are somewhat imprecise and not sensitive to small changes or subtle disorders, which may be indicative of pathology or risk of adverse health events.

In the medium term, new technologies, such as wearable body sensors, will be able to produce more precise data than current clinical methods, and also to monitor changes in gait parameters over time. In addition, they will extend the clinical assessment of gait outside the laboratories to the patients’ home.

However, the lack of good reference patterns, in order to establish what should be considered normal or pathological gait, especially in the elderly population, is a well recognized problem^3^. The few studies on normal gait patterns in the elderly have been conducted in healthy elderly people without comorbidity, and either in laboratory settings or in large clinical facilities^3–9^. Gait varies according to the spaciousness of the place where one is walking^10–12^, so that the normal patterns found in the laboratory can hardly represent the gait older people perform at home, where, paradoxically, they walk most of the time, and thus, where most data of wearable technologies will be collected. In addition, a considerable proportion of the elderly population may have difficulties leaving home due to health, mobility or comfort issues. This implies that the studies on the elderly population conducted away from home (where patients have to commute), may present a selection bias, leaving oldest people or those with the poorest walking ability out of the sample. As a consequence, once more, they may not describe the real gait of the elderly population.

Thus, it is necessary to develop gait studies at home, in order to establish patterns of normality, in different populations, especially in the elderly. Unfortunately, today, technological media based on inertial sensors (such as accelerometers) are not sufficiently reliable and valid to create reference patterns with them^3^. Therefore, there is the need to establish reference patterns of gait, in real environments such as the home, through the use of precise techniques, similar to those of the laboratories. Only this way, we will be able to use new technologies for gait analysis in real live conditions.

To contribute to this unmet need, this study pursues to create reference patterns for the length of the stride and the step width of the Spanish elderly population, when walking short distances at home. In addition, we aim to analyze the relationship between the spatial gait parameters and other mobility or functional variables, which has been seldom studied.

## Methods

This transversal study was conducted on a sample of 431 non-institutionalized persons older than 64 years, who were living in Spain. The sample was collected by multistaged probabilistic sampling. In the first stage of sampling, towns were selected from previously established geographic locations, including differently sized towns from each area (<10,000 habitants; 10,000–1,000,000 habitants or >1,000,000 habitants). In the next stages, districts were then randomly selected from each town and homes were finally selected from each district, by using a mixed sampling procedure: door-to-door and telephone contact. Using the estimations of the National Health Survey 2007, the sample was stratified to maintain proportions of sex and size of the habitat (town), similar to those of the reference population. The sample also included a non-proportional age stratum with over-representation of subjects older than 79 years who, according to the authors’ criterion, best represent the particular physiological characteristics of the elderly population. The study data were collected between years 2007 and 2009.

Data were collected by survey takers. Survey takers received specific training and the field work was thoroughly supervised, as well as the survey questionnaires, codification process and data processing procedure. To minimize the lack of response, all aspects related with the questionnaire and the survey takers were carefully prepared. A candidate was only substituted by another candidate after 10 failed attempts to contact, 2 missed appointments, rejection to participate, institutionalization or death. The response rate was 63%.

To ensure the quality of the field-work a supervisor accompanied the survey takers during the first few visits and a telephonic control was carried out on 15% of participants; they were asked about the sampling procedure (how they had been contacted), the completion of the protocol and the veracity of answers. The fulfilled questionnaires were reviewed by a specially constituted team, which was also in charge of recovering missing data by telephone, whenever possible. Such quality control revealed selection errors for 100 participants, who were not included in the study.

Sociodemographic data were recorded (age, sex, education level, cohabitation status), as well as the list of medicines (medicines used by every participant were recorded) and the list of previous diseases, according to the questionnaire of the National Health Survey 2006^13^. Cognitive capacity was measured with the Pfeiffer’s test^14^ and depression was screened by using the GDS-5^15^. Functional capacity was assessed with the Katz index^16^, which measures the degree of dependence for the following basic activities of daily living (BADL): bathing, dressing, toileting, transferring, continence, and feeding (the index was scored from 0 to 6; 0 for total independence and 6 dependence for all activities). Physical activity was measured by using the following question from the PASE^17^: Over the past 7 days, how often did you take a walk outside your home or yard for any reason? (rated according the scale instructions). All the participants were weighed and their height was measured, muscular strength was assessed by using the Medical Research Council Scale^18^ and balance was assessed by using the first 4 items of the Tinetti balance scale (balance in a sitting position, ability to stand without assistance, balance in standing position and immediate standing)^2^.

**Figure 1:**
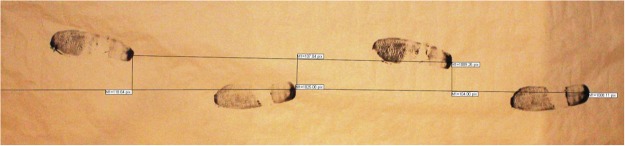
Footprints record.

Spatial gait parameters were recorded at the participants’ homes using an ink footprint record^19,20^, so that the prints could be preserved, transported and later analyzed (Fig. 1). This method consist of spreading brown paper on the floor and make the participants to walk on it at their preferred speed, with the shoes on and the soles impregnated with non-stick washable ink. The paper was extended on the floor of hallways or rooms of the houses. It was 1 meter width, and its length depended on the available space, taking the test in the longest place of the home and registering 4 meters of walking when it was possible. There were no obstacles on the paper path that could hinder the gait.

A digital photograph of the footprints record of each patient was taken with a Canon EOS 1100D camera. In the frame of each photograph, an element of known dimensions was included as a standard measure. This element was a cardboard T, whose arms measured exactly 1 meter. Later, both the spatial gait parameters and the cardboard T were measured in the image in pixels, by using TPS Dig2 software (version 2.16). Real dimensions (in meters) of the spatial gait parameters were obtained by dividing the distance in pixels between footprints in the picture, by the size in pixels of the cardboard T (1 meter). The length of the stride was determined by measuring the distance of the midpoint of the back of the heel, of two consecutive footprints of the same foot. The width of the step was determined by measuring the distance, between the midpoint of the back of the heel, and the line of trajectory of the contra-lateral foot (Fig. 1). The process of recording spatial gait parameters by using the footprints method was video-recorded at home for every participant. Each record was analyzed by two physiotherapists, experienced in this field, who evaluated the correctness of the method, the naturalness of gait and the absence of incidents or artifacts. Only those tests that were approved by both physiotherapists were included in the analysis.

The research was conducted in accordance with the Helsinki Declaration and written informed consent was obtained from all the participants or their legal guardians before being included in the study. The research protocol was previously approved by the local Ethical Committee (*Consorci Sanitari del Maresme*).

### Statistical analysis

Assuming a variance of 0.02 for step length normalized to height, a sample size of 411 was considered enough to make estimations with a maximum error of 0.015, considering also a design effect of 1.2. Moreover, 351 participants were considered sufficient to estimate step width with an error less than 0.5 cm.

To avoid over-representation of any age or sex group, the sample was weighted according to the population in the 2012 annual report of the Spanish National Institute of Statistics (INE).

In all cases the first and last strides given by the subject were excluded from the analysis and the parameters of the rest of the strides were averaged. Stride length and step width were recorded and analyzed in centimeters, as quantitative continuous variables.

Stride length was normalized to subject’s height using the following formula: *normalized stride length* = *stride length (cm)/height (cm)*^21,22^. The resulting value expresses the ratio of the length of the stride to the subject’s height (e.g: a value of 0.5 means that the stride is half part of the individual’s height; a value of 1 represents equivalence between stride length and subject’s height). Also, the ratio of the step width to the stride length was studied by using the following construct: *ratio width to normalized length* = *step width/normalized stride length*. The resulting value is greater when the subject steps are short and wide, and smaller when the subject’s steps are long and narrow.

The kurtosis and symmetry of the variables derived from gait parameters were studied and their adjustment to normal distribution was verified with the Shapiro-Wilks test. The mean, standard deviation, median and interquartile range of gait parameters were calculated. The limits of normality for parameters with normal distribution were defined as a function of the mean and standard deviation: mean +/− 2 SD for the 95% limits, and mean +/− 2.58 SD for the 99% limits. In the case of non-normal variables, the limits of normality were established using percentiles.

Sex and age influence on gait parameters were studied by using ANOVA. The association between the spatial gait parameters and the other health and functional characteristics of the participants was analyzed by using chi-square in the case of categorical variables, and linear regression for quantitative variables.

Finally multivariate models were built to study the joint effect of the relevant variables on gait parameters. For such analysis, a logarithmic transformation of the ratio width to normalized length was made to approximate it to the normal distribution.

All data were analyzed by using R v. 3.3.1 software.

The datasets generated during and analysed during the current study are available from the corresponding author on reasonable request.

## Results

**Figure 2:**
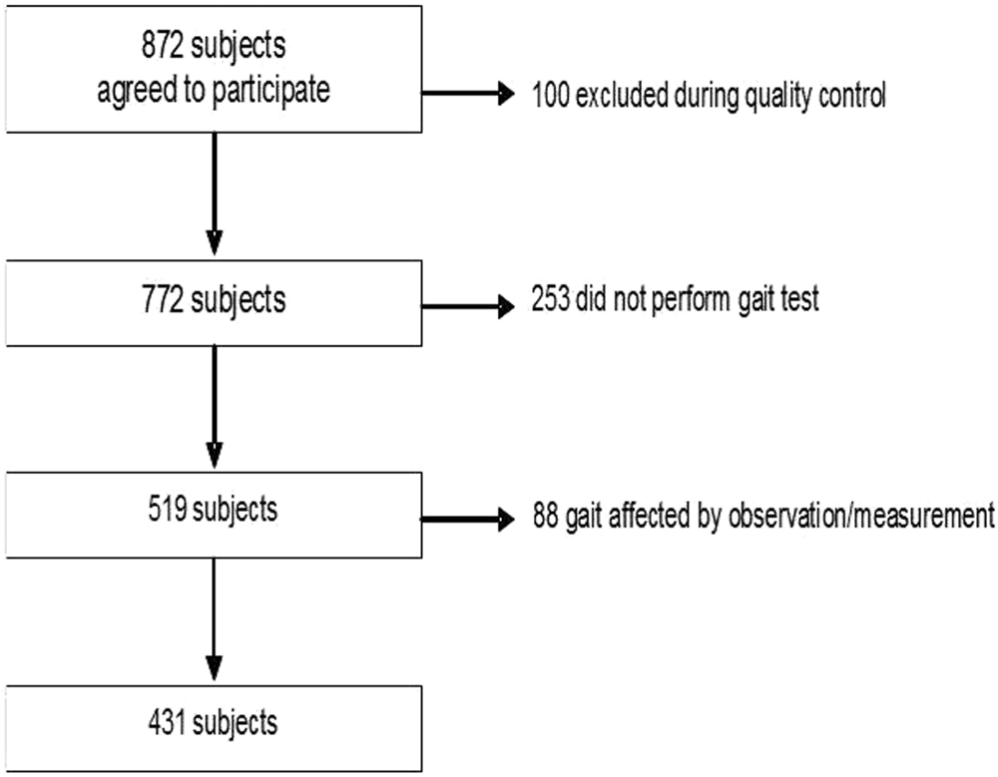
Recruitment and inclusion data.

Of the 772 participants originally selected, 253 were excluded because they could not walk independently or did not feel safe to perform the walking test without assistance. In addition, 88 participants were excluded from the analyzes, because at least one of the two physiotherapists who viewed the videos, considered that their gait was not natural or the method had not been applied correctly. Figure 2 shows a flow diagram of recruitment and inclusion process. The characteristics of the 431 participants finally included in the statistical analysis are shown in Table 1.

**Table 1:** Socio-demographic data of participants who completed the footprint test.

	Mean	SD
Age	76	7.1
Height	159.2	8.8
BMI	28.9	4.5
	**Median**	**IQR**
Comorbidity^a^	3	3
Polypharmacy^a^	4	4
	**n**	**%**
Sex		
Male	184	42.7
Female	247	57.3
Education level		
None	100	23.4
Basic	251	58.7
Intermediate	45	10.4
University	32	7.5
Cohabitation		
Lives alone	109	25.7
Lives with someone	317	74.3
Katz		
0	343	79.7
1–2	81	19
3–4	2	0.5
5–6	4	0.9
Pfeiffer		
0–3	404	94.9
4–7	19	4.6
8–10	3	0.5

SD: Standard deviation.

IQR: Interquartile range.

Stride length, normalized stride length and step width were normal according to the Shapiro-Wilks test, when analyzed within sex and age groups. The ratio width to normalized length, was a non-normal variable (Shapiro statistic 0.87; p < 0.001) with 2.15 asymmetry and 6.03 kurtosis.

After dismissing the first and the last stride, an average of 4 strides were analyzed per person (two from each side). Mean stride length was 88.47 cm (CI95%: 85.52–91.41 cm), SD was 26.05 (CI95%: 24.21–27.76 cm), the average step width was 10.34 cm (CI95%: 9.84–10.83 cm), step width SD was 4.37 (CI95%: 3.94–4.76 cm). Normalized stride length was 0.55 (CI95%: 0.54–0.57), SD: 0.15 (CI95%: 0.14–0.16 cm). Normal limits by sex and age and in the total group are shown in Table 2.

**Table 2:** Normal limits by sex and age of the spatial gait parameters.

	Stride length	Mean	SD	Normal limits (95%)		Normal limits (99%)		N
65–79	Men 65–79	110.7	18.8	73.2	148.24	61.9	160	71
	Women 65–79	87.7	18.5	51	124.7	39.71	135.75	98
>79	Men >79	73.1	25.1	25.9	126	10.8	141.5	98
	Women >79	63.1	20.5	22.2	104.6	9.9	116.4	164
TOTAL	(weighted)	88.5	26.1	36.4	140.6	20.8	156.2	431
	Normalized stride length*	**Mean**	**SD**	**Normal limits (95%)**		**Normal limits (99%)**		**N**
65–79	Men 65–79	0.66	0.11	0.44	0.89	0.37	0.95	71
	Women 65–79	0.57	0.12	0.33	0.8	0.26	0.87	98
>79	Men >79	0.47	0.15	0.16	0.77	0.07	0.86	98
	Women >79	0.41	0.13	0.15	0.68	0.07	0.75	164
TOTAL	(weighted)	0.55	0.15	0.25	0.86	0.16	0.95	431
	**Step width**	**Mean**	**SD**	**Normal limits (95%)**		**Normal limits (99%)**		**n**
65–79	Men 65–79	10	4.5	1.1	19	−1.6	21.6	71
	Women 65–79	9.4	3.8	1.9	17	−0.4	19.2	98
>79	Men >79	12.5	4.9	2.6	22.3	−0.3	25.2	98
	Women >79	11.4	4.5	2.4	20.5	−0.3	23.2	164
TOTAL	(weighted)	10.3	4.37	1.6	19.8	−1	21.7	431
	Ratio width-length**	**Median**	**IQR**	**Normal limits (95%)**		**Normal limits (99%)**		**n**
65–79	Men 65–79	16.2	9.3	1.9	34	1.6	44	71
	Women 65–79	16.1	8.9	4	36.5	0.8	78.7	98
>79	Men >79	28.2	24.7	5.4	87.7	4.1	95.5	98
	Women >79	26.8	26.7	4	82.6	1.1	93.9	164
TOTAL	(weighted)	17.4	15	4.1	68.8	0.9	100	431

^*^Stride length normalized to subject height.

^**^Step width/normalized stride length.

As expected, stride length was correlated to person’s height (Pearson’s r = 0.45; p < 0.001), and was inversely correlated with stride width (Pearson’s r = −0.34; p < 0.001). Step width was not significantly associated with person’s height or BMI.

Stride length was shorter in women and in the older group (p < 0.001). An interaction between sex and age was observed in their effect on the stride length (ANOVA: p of interaction <0.0271). This interaction did not affect the normalized stride length, which also decreases with age and in the female sex (p < 0.001). Step width was larger in the older elders (p < 0.001), although no differences were found between sexes. The effect of age in width and length was markedly appreciated in the ratio width to normalized-length, which was more than double in the oldest old group comparing to the young elders (p < 0.001), not being this index affected by sex (Table 2).

The bivariate analysis showed that normalized stride length was also associated to person’s strength (p < 0.001), balance (p < 0.001), functional capacity (p < 0.001), cognitive capacity (p < 0.001), affective status (depression:p < 0.001 and anxiety: p < 0.001), number of drugs (p < 0.06) and physical activity (frequency of walking outdoors; p < 0,001). Step width was associated to person’s strength (p < 0.001), balance (p < 0.001), functional capacity (p < 0.01) and anxiety: p < 0.001). Finally, the ratio width to normalized-length resulted associated to strength (p < 0.001), balance (p < 0.001), functional capacity (p < 0.001), cognitive capacity (p < 0.01), affective status (depression:p < 0.001 and anxiety: p < 0.01), and physical activity (frequency of walking outdoors; p < 0,01).

Table 3 shows the results of the multivariate analysis conducted including gait parameters and physical and health variables. While stride length was affected by a number of factors, step width resulted only influenced by balance. The ratio width to normalized length, was mainly influenced by age and strength, accounting both variables for 21.4%of the variability of this composed gait parameter (Mc Fadden’s R^2^).

**Table 3:** Results of multivariable models.

	Variable	Estimator	IC95%		p
Normalized stride length*	Muscle strength	0,01	0.004	0.012	**<0**,**001**
	Balance	0,02	0.007	0.033	**0**,**003**
	Age	−0,01	−0.010	−0.006	**<0**,**001**
	Sex (male)	0,07	0.037	0.099	**<0**,**001**
	Physical activity: frequent	0,04	0.001	0.079	**0**,**047**
	Physical activity: never	−0,04	−0.115	0.034	0,291
	Physical activity: rare	0,05	0.053	−0.003	0,065
	Depression	−0,01	−0.011	−0.021	**0**,**04**
	**Variable**	**Estimator**	**IC95%**		
Step width	Balance	−0,79	−1.234	−0.346	**<0**,**001**
	**Variable**	**Estimator**	**IC95%**		
Ratio width-length**	Muscle strength	−0.06	−0.070	−0.041	**<0**,**001**
	Age	0.027	0.016	0.038	**0**,**003**

Bold type: significant finding.

^*^Stride length normalized to subject height (Estimator is based in changes of 0.1 units of normalized stride length).

^**^Step width/normalized stride length.

## Discussion

In this article, we report the normal limits of spatial gait parameters in older adults, as measured in a short walk at home, and we study their association with other health variables.

The stride length is related to the subject’s height and the step width is inversely related to the stride length. Taking these data into account, we normalized stride length by height, and developed a new index that represents the ratio between the width of the step and the normalized stride length.

Stride length is shorter in the group of older elders and in women while step width gets larger with age and is not affected by sex. Positive interactions were detected in the influence of sex and age on stride length, but not in their influence on normalized stride length. This could mean that sex-age interactions are mediated by individual’s height, and highlights the importance to normalize the stride length by subjects’ height or by leg’s length. Some authors do not support the normalization of the stride length, because of the difficulty of interpreting its results^23^. We believe however, that the comparison of the stride length with subject’s height is a rather intuitive measure, considering that in the majority of the elderly, the stride length is slightly longer than half of their height.

The spatial parameters of gait have been measured by other authors in several studies. However, we failed to find studies measuring gait parameters in real conditions, at home. Hollman *et al*. measured the gait parameters of 249 elders (older than 70 years) in a research center, and found 131 ± 17 cm stride length for men older than 80 years (n 37) and 111 ± 14 cm for women older than 80 years (n 43). Stride length was shorter for subjects older than 85 years: 119 ± 21 for men (n 14) and 109 ± 18 for women (n 33). In this study, the average step width ranged from 7.9 cm to 11.2 cm for these sex and age groups^4^. Verlinden *et al*. reported an average stride length of 129.8 cm (SD 17 cm) and a width of 10.1 cm (SD 4.0) in a sample of 1500 subjects older than 60 years in the Netherlands (average height 168.5 cm)^6^. On the other hand, Oh-Park *et al*. reported shorter mean stride length (109.9 cm; SD 20.2) in a sample of 834 subjects older than 70 years in the United States. They also found a clear reduction in the stride length depending on subjects’ age and sex, with 98.8 cm for women older than 85 years^7^. Thaler-Kall *et al*. found a 117.2 cm (SD 17.8 cm) stride length and a 9.15 cm (SD 3.26 cm) step width in the German elders^8^. All of these studies were carried out with the *electronic walkway* GAITRite®^24^, in gait laboratories or spacious facilities of clinical or research centers. Finally, Beauchet *et al*.^3^, who were aware of the lack of reference patterns for elderly gait, merged databases from the GOOD initiative^25^ and the Generation 100 study^26^, to obtain normative gait parameters of the elderly population. Again, their data were not collected at home and length of the stride they found, was larger than ours. Interestingly, they only had data of 36 patients older than 85 years, which pose a limitation they explicitly recognized, along with the need of further efforts to explore the gait of this oldest group, which is the fastest growing age group, and has the highest prevalence and of gait disorders.

The step width in our study was within the ranges reported by other authors. However, our mean stride length was shorter than in any other paper. It could be postulated that such a reduced stride length could be related to our method of printing the steps with ink. In our opinion, it is improbable since talc and ink footprints records are classic validated methods widely used before the development of the modern electronic walkways^19,20,27–30^, and which have occasionally served as a comparison standard for these^31^. Moreover, we were particularly careful excluding from the sample all gait records suspected unnatural or artifactual due to the method. We propose that the short stride lengths in our study could result from the fact that gait was measured in a short walk at home, from the advanced mean age of our sample (more than half of the sample were older than 79 years) or from the lower mean height of the Spanish population as compared to other countries, where other studies were conducted. Measuring the gait at home is particularly interesting, since no one was excluded for failing in moving to the lab, and due to the potential application of results to the clinical practice, where measuring gait parameters in wide, spacious gait laboratories is often not possible. Problems derived from comparing populations with different mean heights could be solved by comparing normalized stride lengths. However, even when the stride length depends largely on the subject’s height, normalized stride length has seldom been reported in studies. Among the above mentioned ones, only Thaler-Kall *et al*. reported normalized stride length values. However – although the authors did not make it clear – they were probably normalized by the length of the leg, instead of the subject’s height; thus they are not comparable to our data.

In our study we have observed that stride length, in addition to being influenced by age and sex, is related to physical activity, balance and strength, whereas the width of the step seems to be only associated with balance. Interestingly, depression remained in the final model for normalized stride length, weakly associated with it. This is not surprising, as depressed patients’ gait characteristics have been found to differ clearly from normal controls, and to improve with depression treatment^32^. We did not find other papers studying the relationship between step width or stride length and mobility or function variables, such as strength, balance, physical activity or functional capacity, in older adults. It is interesting to mention however, the study by Taniguchi *et al*. in which the authors demonstrated, in a large longitudinal study, that a shorter stride length (measured repeated over time) was associated with higher risk of incident disabling dementia (Hazzar Ratio: 2.12 for usual pace and 2.8 for maximum pace)^33^.

The ratio width to normalized length represents how wide the steps in proportion to its length are. This composite parameter normally takes low values, since normal walking implies narrow and long steps, while gait or balance disturbances may cause steps to be wider, shorter, or both. As stride length is affected by height (taller individuals walk with larger strides), we included the normalized-to-height stride length in the new parameter, to avoid the effect of the height in the composite parameter. As a consequence, the measuring units of this parameter are cm^-1^, since normalized stride length has no units.

It is worth noting that the ratio width to normalized length, has shown to be markedly different between age groups, representing well the global changes of age in the spatial gait parameters (senile gait). This parameter was associated with muscle strength as well, so it can be a good marker of fragility, a hypothesis that will have to be investigated in future studies.

Our initial response rate was not particularly low, given the characteristics of the study, and it was in line with similar studies^34^. However, besides the initial lack of response, some patients were excluded because they could not walk without help and others due to our thorough quality control of the gait test, where all records suspected artifactual were disregarded. All the losses together were considerable; thus, the generalization of our observations could be compromised, which constitutes a limitation. However, the sample size was large enough to estimate gait parameters reasonably accurately and to find significant associations with other factors. It should also be taken into account that conducting studies including the step width is laborious and/or requires technological means; thus, in spite of the sample reduction, our study is still one of the largest ones published in this field.

In conclusion, the normal stride length of elders at home ranges from 36 to 141 cm and width ranges from 1.6 to 20 cm. Older elders have a markedly wider step in proportion to their normalized stride length comparing to younger elders. The spatial parameters of gait are mainly influenced by the subjects’ strength and balance.
